# Evaluation of a population-based breast cancer screening in North China

**DOI:** 10.1007/s00432-023-04905-w

**Published:** 2023-06-02

**Authors:** Siqi Wu, Di Liang, Jin Shi, Daojuan Li, Yanyu Liu, Yahui Hao, Miaomiao Shi, Xinyu Du, Yutong He

**Affiliations:** Cancer Institute, The Fourth Hospital of Hebei Medical University and Hebei Tumor Hospital, No. 12 Jian Kang Road, Changan District, Shijiazhuang, 050011 Hebei China

**Keywords:** Screening, Breast cancer, Detection rate, Participation rate, Hebei province

## Abstract

**Background:**

Despite mammography-based screening for breast cancer has been conducted in many countries, there are still little data on participation and diagnostic yield in population-based breast cancer screening in China.

**Methods:**

We enrolled 151,973 eligible women from four cities in Hebei Province within the period 2013–2021 and followed up until December 31, 2021. Participants aged 40–74 who assessed as high risk were invited to undergo breast ultrasound and mammography examination. Overall and group-specific participation rates were calculated. Multivariable analyses were used to estimate the factors associated with participation rates. The diagnostic yield of both screening and no screening groups was calculated. We further analyzed the stage distribution and molecular subtype of breast cancer cases by different modes of cancer detection.

**Results:**

A total of 42,547 participants were evaluated to be high risk of breast cancer. Among them, 23,009 subjects undertook screening services, with participation rate of 54.08%. Multivariable logistic regression model showed that aged 45–64, high education level, postmenopausal, current smoking, alcohol consumption, family history of breast cancer, and benign breast disease were associated with increased participation of screening. After median follow-up of 3.79 years, there were 456 breast cancer diagnoses of which 65 were screen-detected breast cancers (SBCs), 27 were interval breast cancers (IBCs), 68 were no screening cancers, and 296 were cancers detected outside the screening program. Among them, 92 participants in the screening group (0.40%) and 364 in the non-screening group (0.28%) had breast cancer detected, which resulted in an odds ratio of 1.42 (95% CI 1.13–1.78; *P* = 0.003). We observed a higher detection rate of breast cancer in the screening group, with ORs of 2.42 (95% CI 1.72–3.41) for early stage (stages 0–I) and 2.12 (95% CI 1.26–3.54) for luminal A subtype. SBCs had higher proportion of early stage (71.93%) and luminal A subtype (47.22%) than other groups.

**Conclusions:**

The significant differences in breast cancer diagnosis between the screening and non-screening group imply an urgent need for increased breast cancer awareness and early detection in China.

## Introduction

Breast cancer is the most common malignancy and primary cause of cancer mortality among women globally, with an estimated 2.26 million new cases and 685,000 new deaths in 2020 (Sung and Siegel 2021). It is estimated that 420,000 breast cancer new cases were diagnosed and 120,000 deaths in China in 2020, accounting for 18.4 and 17.1% of all the world cases (Lei et al. 2021). Our understanding of breast cancer etiology and prognosis has improved over time, and the treatment outcome and survivorship can be improved through earlier detection.

Mammography screening has been demonstrated to reduce breast cancer mortality (Canelo-Aybar et al. 2022; Xie et al. 2022; Moss et al. 2015). Nevertheless, the participation remains low in China due to health check-up hesitancy toward cancer screening and low cancer awareness to make informed choices in the target population (Cao et al. 2021). A population-based study in China enrolled 313,022 high-risk individuals of breast cancer during the period of 2013–2017, showing that a participation rate was 40.3% (Chen et al. 2020). Previous studies have shown that participation rates differ significantly between different populations (Mottram et al. 2021; Wu et al. 2019). Additionally, cultural factors, the health care system's infrastructure, and economic concerns also influence the effectiveness of organized population-based screening (Williams et al. 2015). Results from other countries may not apply to China because of different cultural background and delivery of screening. Therefore, it is urgent to explore the participation and possible determinants of cancer screening in China.

Stage distribution and molecular subtypes are one of the most important factors affecting prognosis. Studies showed that majority of screen-detected breast cancers (SBCs) were small in size, node-negative, of early stages and positive for estrogen receptor (ER) and progesterone receptor (PR) (Bellio et al. 2017; Johnson et al. 2019). Other studies observed no differences in molecular subtype between SBCs and interval cancers (IBCs), emphasizing the need for additional study (Irvin et al. 2020). A few studies examined breast cancer molecular subtypes according to cancer detection mode, and the relationship between them is not well understood (Cheasley et al. 2019; Farshid and Walters 2018). The distribution of breast cancer stage at diagnosis and molecular subtype has not been reported based on a large multi-center population-based screening program in China. Besides, Chinese women tend to be denser breasts (Sung et al. 2018), and have an average age at diagnosis of 45–55 years, much earlier than most western countries (Sun et al. 2018). Understanding the diagnostic yield of breast cancer screening in China may provide scientific evidence for disease prevention and early detection.

The main aim of this study was to understand the participation and diagnostic yield of breast cancer screening program in Hebei within the period 2013–2021. In addition, we analyzed the stage distribution and molecular subtype of breast cancer cases based on different cancer detection methods, so as to provide evidence support for the optimization of breast cancer screening strategies in China.

## Methods

### Study design and population

In 2012, China launched the Cancer Screening Program in Urban China (CanSPUC) (Wang et al. 2019). Hebei Province initiated and conducted the screening program for breast cancer as one of the first eight regions in China. This multi-center population-based study was under CanSPUC within the period 2013–2021, which covered four cities (Shijiazhuang, Tangshan, Xingtai, and Handan) in Hebei Province. In brief, researchers approached residents living in selected neighborhoods in participating cities by telephone and personal encounter. The target population was required to be women without a history of cancer who lived local for 3 years, aged 40–74. Qualified participants were assessed for cancer risk by a defined risk score system. Trained staff interviewed all eligible participants to determine their risk factors’ exposure. Only women identified as high risk for breast cancer were invited to receive breast ultrasound and mammography examination in a designated tertiary hospital. The Fourth Hospital of Hebei Medical University Ethics Board approved the study and written informed consent was obtained from all participants prior to implementation.

### Risk assessment

High-risk individuals were identified using the Harvard Cancer Risk Index (Colditz et al. 2000). The risk scoring system for breast cancer included the following factors: age, body mass index (BMI), marital status, education, smoking history, alcohol consumption, breastfeeding, menopause status, family history of breast cancer, and benign breast disease. The expert panel assigned coefficient scores for each risk factor according to its correlation with breast cancer (He Yutong et al. 2021). The final individual relative risk is calculated by divided the cumulative risk score from population's mean risk score.

### Clinical procedures

High-risk participants were referred for breast ultrasound and mammography in the tertiary-level hospital. All examinations were performed by radiologists who had at least 5 years of experience. Clinical information was recorded concerning mass characteristics, asymmetry density, calcification, and architectural distortion. The largest lesion was recorded if the subject has more than one lesion in the breast (multifocal). The reporting standard in both examinations was the Breast Imaging Reporting and Data System (BI-RADS) classification (Rao et al. 2016).

Annual capacity training was conducted across all study sites to ensure that radiologists are performing BI-RADS to the uniform standard. For each examination, experts from the National Cancer Center reviewed images of 1% of randomly selected negative results and all positive results (BI-RADS categories of 3, 4, and 5). Any differences from the original diagnosis were discussed until an agreement was reached.

### Follow-up data

Women who screened with positive results were followed up by telephone or retrieval of medical record information to obtain the final diagnosis and outcome. The entire cohort population was passively followed up using the population-based Hebei Cancer Registry Database from October 1, 2013 until December 31, 2021. Tumor characteristics (including stage at diagnosis, molecular subtypes, and histological types) were collected from pathological reports by trained investigators. All breast cancer cases were reported according to the International Statistical Classification of Disease, Tenth Revision (codes D05.0-D05.9 and C50.0-C50.9).

Screen-detected breast cancer (SBC) referred to cancer that is identified within 0–6 months after a positive screening result. Interval breast cancer (IBC) was defined as cancer that was detected between 0 and 24 months after a negative screening result (Niraula et al. 2020). Women who were high risk for breast cancer but did not undergo any screening are classified as noncompliant breast cancers (NBCs). Breast cancer identified in women who had a low risk of developing the disease were labeled as cancers detected outside of the screening program.

The stage at diagnosis was categorized based on the American Joint Committee on Cancer Staging (AJCC) 7th edition (Edge and Compton 2010). We defined early stage using stages 0 to I. We extracted detailed information on status of ER, PR, human epidermal growth factor receptor 2 (HER 2), and Ki67 status from the pathological reports. Results of 1% or more tumor nuclear of positive staining were classed as positive ER (ER^+^) or PR (PR^+^) (Allison et al. 2020). Positive HER2 (HER2^+^) was defined as positive nuclear staining intensity in "2+" and "3+" of tumor cells (Wolff et al. 2018). The molecular subtype was classified according to the 2013 St. Gallen criteria (Zhang et al. 2019).

### Statistical analysis

We presented the characteristics of the study population, and overall and group-specific participation by categorical variables. Chi-squared test was used to compare the association between candidate variables and participation. We further explored the potential factors associated with participation in breast cancer screening. Odds ratio (OR) and 95% confidence intervals (CIs) were estimated by logistic regression. Diagnostic yield among different groups, including detection rates of stage at diagnosis, histological type, and molecular subtype of breast cancer, was calculated. We further analyzed the stage distribution and molecular subtype of breast cancer cases by different cancer detection modes. We used R software (version 4.1.2) for all analyses, and considered *P* values of 0.05 or less to be statistically significant. All hypotheses were two-sided.

## Results

### Characteristics of the study population

**Fig. 1:**
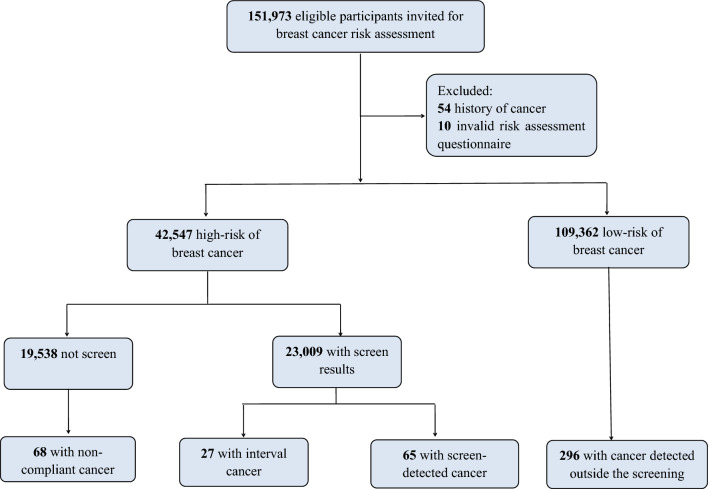
The flowchart of participants included in the analyses

Overall, 151,973 eligible participants were recruited from 2013 to 2021. After excluding participants assessed as low risk for breast cancer (*N* = 109,362), those with history of cancer (*N* = 54), and ineffective risk assessment results (*N* = 10), 42,547 participants were identified as high risk for breast cancer (Fig. 1).

Characteristics of high-risk women and who underwent screening are shown in Table 1. The majority (78.1%) of participants were 45–64 years, with the average age of 54.57 years (SD = 8.2 years). In addition, 59.1% of the high-risk group was overweight or obese, 52.6% had a family history, and 68.7% had one or more benign diseases. Among the 42,547 high-risk individuals, 23,009 participants underwent screening, with participation rate of 54.08%. In total, Handan had the highest participation rate (67.7%), compared with the lowest participation rate in Xingtai (50.5%).

**Table 1:** Characteristics of high-risk populations and participation rates

Factors	High risk for breast cancer, *n* (%)	Undertaking screening, *n* (%)	Participation rate, %	*P* values^a^
*City*				
Shijiazhuang	13,241 (31.1)	8110 (35.2)	60.43	< 0.001
Tangshan	26,210 (61.6)	13,281 (57.7)	50.67	
Xingtai	2780 (6.5)	1404 (6.1)	50.50	
Handan	316 (0.7)	214 (0.9)	67.72	
*Years*				
2013–2014	4900 (11.5)	1800 (7.8)	36.73	< 0.001
2014–2015	4536 (10.7)	1939 (8.4)	42.75	
2015–2016	3391 (8.0)	1886 (8.2)	55.62	
2016–2017	5003 (11.8)	3147 (13.7)	62.90	
2017–2018	4712 (11.1)	3015 (13.1)	63.99	
2018–2019	5974 (14.0)	3470 (15.1)	58.09	
2019–2020	5324 (12.5)	3795 (16.5)	71.28	
2020–2021	8707 (20.5)	3957 (17.2)	45.45	
*Age (years)*				
40–44	4624 (10.9)	2201 (9.6)	47.60	< 0.001
45–49	8753 (20.6)	4587 (19.9)	52.40	
50–54	8868 (20.8)	5166 (22.5)	58.25	
55–59	7595 (17.9)	4458 (19.4)	58.70	
60–64	6746 (15.9)	3760 (16.3)	55.74	
65–69	4516 (10.6)	2278 (9.9)	50.44	
70–74	1445 (3.4)	559 (2.4)	38.69	
*BMI (kg/m*^*2*^*)*				
< 18.5	522 (1.2)	250 (1.1)	47.89	0.017
18.5–	16,897 (39.7)	9161 (39.8)	54.22	
≥ 24.0–	25,128 (59.1)	13,598 (59.1)	54.11	
*Marital status*				
Married	40,311 (94.7)	21,879 (95.1)	54.28	0.001
Other^b^	2236 (5.3)	1130 (4.9)	50.54	
*Education level*^c^				
Low	5241 (12.3)	2420 (10.5)	46.17	< 0.001
Intermediate	28,842 (67.8)	15,794 (68.6)	54.76	
High	8464 (19.9)	4795 (20.8)	56.65	
*Menopause status*				
Premenopausal	16,517 (38.8)	8356 (36.3)	50.59	< 0.001
Postmenopausal	26,030 (61.2)	14,653 (63.7)	56.29	
*Smoking history*				
Never	31,747 (74.6)	16,243 (70.6)	51.16	< 0.001
Current	10,159 (23.9)	6384 (27.7)	62.84	
Former	641 (1.5)	382 (1.7)	59.59	
*Alcohol consumption*				< 0.001
Never	32,889 (77.3)	16,928 (73.6)	51.47	
Current	8934 (21.0)	5640 (24.5)	63.13	
Former	724 (1.7)	441 (1.9)	60.91	
*Age at menarche, years*				
< 12	30,915 (72.7)	16,762 (72.9)	54.22	0.355
≥ 12	11,625 (27.3)	6244 (27.1)	53.71	
*Breastfeeding*				
No	6412 (16.1)	3629 (16.2)	56.60	0.651
Yes	33,336 (83.9)	18,762 (83.8)	56.28	
*Benign breast diseases*				
No	12,648 (31.3)	5737 (26.5)	45.36	< 0.001
Yes	27,815 (68.7)	15,947 (73.5)	57.33	
*Family history of breast cancer*				
No	20,177 (47.4)	9338 (40.6)	46.28	< 0.001
Yes	22,370 (52.6)	13,671 (59.4)	61.11	
Total	42,547	23,009	54.08	

BMI, Body mass index

^a^*P* values were calculated using Chi-square test

^b^Other: including the unmarried, divorced, or widowed

^c^Education level: low: primary school or below; intermediate: junior or senior high school; high: undergraduate or over

### Factors associated with screening participation

In univariate analyses, women aged 45–69 years; married; postmenopausal; current smoking; alcohol consumption; high level of education; with benign disease and family history were more likely to participate in the study. To explore the potential factors associated with participation rate, we also conducted multivariable logistic regression models (Table 2**)**. We found that participation rate was associated with age, education level, current smoking, alcohol consumption, menopause status, benign breast disease, and family history of breast cancer. For instance, women with benign breast disease had 30% greater likelihood of undertaking screening than those without (OR = 1.30, 95% CI 1.24–1.37). The odds of participants with family history undergoing screening were 29% higher odds than participants without (OR = 1.29, 95% CI 1.23–1.35). While women aged 70–74 were less likely to undergo screening compared to those aged 40–45 (OR = 0.68, 95% CI 0.58–0.78). We additionally adjusted the study sites and recruitment year in the model II, and the odds ratios did not change significantly.

**Table 2:** Factors associated with participation rate in breast cancer screening

Factors	Model I^a^			Model II^b^	
	OR (95%CI)	*P* values		OR (95%CI)	*P* values
*Age (years)*					
40–44	Reference			Reference	
45–49	1.19 (1.11 to 1.28)	< 0.001		1.23 (1.14 to 1.33)	< 0.001
50–54	1.40 (1.29 to 1.52)	< 0.001		1.41 (1.30 to 1.54)	< 0.001
55–59	1.41 (1.29 to 1.55)	< 0.001		1.43 (1.30 to 1.58)	< 0.001
60–64	1.28 (1.17 to 1.41)	< 0.001		1.28 (1.16 to 1.41)	< 0.001
65–69	1.10 (1.00 to 1.22)	0.060		1.10 (0.99 to 1.22)	0.084
70–74	0.75 (0.65 to 0.87)	< 0.001		0.68 (0.58 to 0.78)	< 0.001
*BMI (kg/m*^*2*^*)*					
18.5–23.9	Reference			Reference	
< 18.5	0.85 (0.71 to 1.03)	0.089		0.89 (0.74 to 1.07)	0.220
≥ 24.0	1.02 (0.98 to 1.06)	0.392		1.03 (0.98 to 1.07)	0.260
*Education level*					
Low	Reference			Reference	
Intermediate	1.31 (1.23 to 1.40)	< 0.001		1.32 (1.24 to 1.41)	< 0.001
High	1.51 (1.40 to 1.63)	< 0.001		1.50 (1.38 to 1.62)	< 0.001
*Marital status*					
Married	Reference			Reference	
Others	0.99 (0.90 to 1.08)	0.753		0.96 (0.87 to 1.05)	0.314
*Smoking history*					
Never	Reference			Reference	
Current	1.24 (1.17 to 1.31)	< 0.001		1.41 (1.32 to 1.49)	< 0.001
Former	1.10 (0.93 to 1.30)	0.252		1.20 (1.01 to 1.42)	0.038
*Alcohol consumption*					
Never	Reference			Reference	
Current	1.24 (1.17 to 1.32)	< 0.001		1.19 (1.13 to 1.27)	< 0.001
Former	1.33 (1.14 to 1.55)	< 0.001		1.27 (1.09 to 1.50)	0.003
*Menopause status*					
No	Reference			Reference	
Yes	1.25 (1.18 to 1.33)	< 0.001		1.27 (1.20 to 1.35)	< 0.001
*Benign breast disease*					
No	Reference			Reference	
Yes	1.30 (1.24 to 1.37)	< 0.001		1.07 (1.02 to 1.13)	0.006
*Family history of breast cancer*					
No	Reference			Reference	
Yes	1.29 (1.23 to 1.35)	< 0.001		1.18 (1.13 to 1.24)	< 0.001

OR, odds ratio; BMI, Body Mass Index

^a^ORs were adjusted for factors including age, BMI, marital status, education, menopause, benign breast disease, and family history of breast cancer in the logistic regression model

^b^Except for the factors included in model I, ORs were additionally adjusted for year of recruitment and study sites in logistic regression model

### Follow-up results

**Fig. 2:**
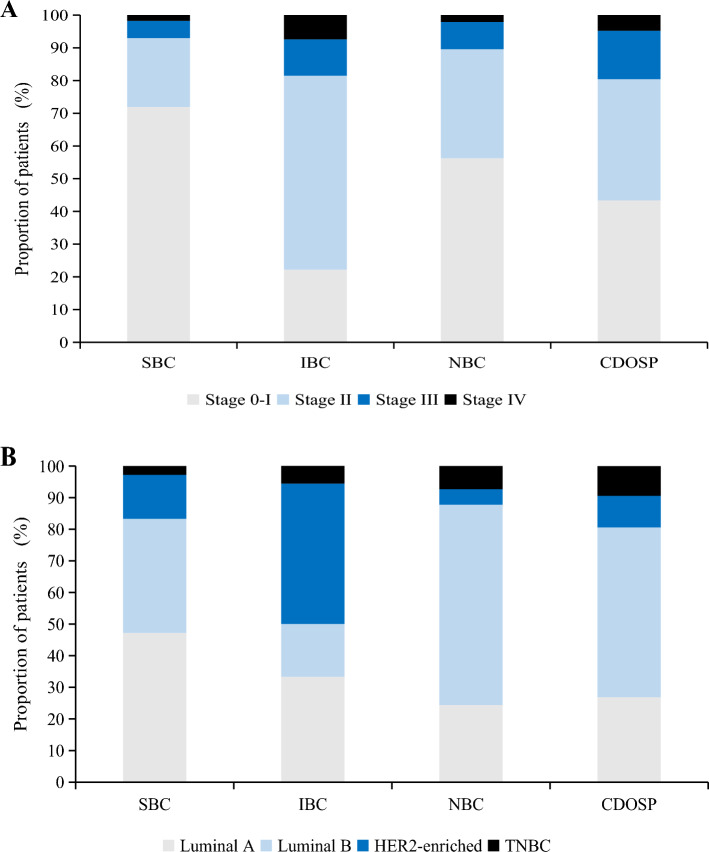
Stage distribution and molecular subtype of breast cancer by cancer detection methods

After a median time of 3.79 year follow-up, there are overall 456 breast cancer diagnoses of which 65 were screen-detected breast cancers (SBCs), 27 were interval breast cancers (IBCs), 68 were noncompliant breast cancers (NBCs), and 296 were cancers detected outside the screening program, yielding the detection rates for SBCs, IBCs, NBCs, and cancers detected outside the screening program at 0.28%, 0.12%, 0.35%, and 0.27%, respectively (Table 3). Of 321 patients with known stage, SBCs had the highest proportion of early stage (stages 0–I) (71.93%), followed by NBCs (56.25%), cancers detected outside the screening program (43.39%), and IBCs (22.22%) (Fig. 2A). Of 255 patients with known molecular subtype, the percentage of HER2-enriched and triple-negative subtype accounted for 50% of the IBCs, 19.38% of the cancers detected outside the screening program, 16.67% of the SBCs, and 12.2% of the NBCs (Fig. 2B).

**Table 3:** Diagnostic yield of breast cancer in this screening program until December 31, 2021

Characteristics	Screen-detected cancer (*n* = 65)	Interval cancer (*n* = 27)	Noncompliant cancer (*n* = 68)	Cancers detected outside the screening program (*n* = 296)
*Age (years)*				
40–44	6 (0.03)	1 (0.00)	5 (0.03)	25 (0.02)
45–49	12 (0.05)	8 (0.03)	18 (0.09)	35 (0.03)
50–54	12 (0.05)	11 (0.05)	10 (0.05)	44 (0.04)
55–59	7 (0.03)	2 (0.01)	13 (0.07)	64 (0.06)
60–64	17 (0.07)	2 (0.01)	11 (0.06)	67 (0.06)
65–69	9 (0.04)	3 (0.01)	7 (0.04)	40 (0.04)
70–74	2 (0.01)	0 (0.00)	4 (0.02)	21 (0.02)
*Stage at diagnosis*				
0–I	41 (0.18)	6 (0.03)	27 (0.14)	82 (0.07)
II	12 (0.05)	16 (0.07)	16 (0.08)	70 (0.06)
III	3 (0.01)	3 (0.01)	4 (0.02)	28 (0.03)
IV	1 (0.00)	2 (0.01)	1 (0.01)	9 (0.01)
Unknown	8 (0.03)	0 (0.00)	20 (0.10)	107 (0.10)
*Histological type*				
Ductal	43 (0.19)	17 (0.07)	38 (0.19)	159 (0.15)
Lobular	2 (0.01)	0 (0.00)	0 (0.00)	6 (0.00)
Others	4 (0.02)	3 (0.01)	4 (0.02)	17 (0.02)
Unknown	16 (0.07)	7 (0.03)	26 (0.13)	114 (0.10)
*Molecular subtype*				
Luminal A	17 (0.07)	6 (0.03)	10 (0.05)	43 (0.04)
Luminal B	13 (0.06)	3 (0.01)	26 (0.13)	86 (0.08)
HER2-enriched	5 (0.02)	8 (0.03)	2 (0.01)	16 (0.01)
Triple-negative	1 (0.00)	1 (0.00)	3 (0.02)	15 (0.01)
Unknown	29 (0.13)	9 (0.04)	27 (0.14)	136 (0.12)
Total	65 (0.28)	27 (0.12)	68 (0.35)	296 (0.27)

The tumor characteristics of breast cancer stage, histologic type, and molecular subtype by screening status are summarized in Table 4. The detection rate of breast cancer was 0.40% (92/23009) in the screening women and 0.28% (364/128900) in the non-screening women, and the OR was 1.42 (95% CI 1.13–1.78; *P* = 0.003). For stage at diagnosis, we observed a higher detection rate of breast cancer in the screening group, with ORs of 2.42 (95% CI 1.72–3.41) for stage 0–I and 1.83 (95% CI 1.19–2.80) for stage II. For histological type and molecular subtype, we observed a higher detection rate of breast cancer in the screening group, with ORs of 1.71 (95% CI 1.28–2.28) for ductal type and 2.12 (95% CI 1.26–3.54) for luminal A subtype.

**Table 4:** Breast cancer stage, histologic type and molecular subtype by screening status

Variable	Group, no. (%)			
	Screening (*n* = 92)	Nonscreening (*n* = 364)	OR (95%CI)	*P* value
*Tumor stage*				
0–I	47 (0.21)	109 (0.08)	2.42 (1.72–3.41)	< 0.001
II	28 (0.12)	86 (0.07)	1.83 (1.19–2.80)	0.006
III	6 (0.03)	32 (0.02)	1.05 (0.44–2.51)	0.912
IV	3 (0.01)	10 (0.01)	1.68 (0.46–6.11)	0.430
Unknown	8 (0.03)	127 (0.10)	0.35 (0.17–0.72)	0.004
*Histological type*				
Ductal	60 (0.26)	197 (0.15)	1.71 (1.28–2.28)	< 0.001
Lobular	2 (0.01)	6 (0.00)	1.87 (0.38–9.25)	0.44
Others	7 (0.03)	21 (0.02)	1.87 (0.79–4.39)	0.15
Unknown	23 (0.10)	140 (0.11)	0.92 (0.59–1.43)	0.71
*Molecular subtype*				
Luminal A	23 (0.10)	53 (0.04)	2.12 (1.26–3.54)	0.004
Luminal B	16 (0.07)	112 (0.09)	0.80 (0.47–1.35)	0.404
HER2-enriched	13 (0.06)	18 (0.01)	1.87 (0.74–4.71)	0.185
Triple-negative	2 (0.01)	18 (0.01)	0.62 (0.14–2.68)	0.525
Unknown	38 (0.17)	163 (0.13)	1.65 (1.20–2.28)	0.002
Total	92 (0.40)	364 (0.28)	1.42 (1.13–1.78)	0.003

OR, odds ratio

## Discussion

This study reported the results of 151,973 participates underwent breast cancer screening from 2013 to 2021 in China. Our results suggest that the detection rate and early diagnosis rates were higher in the screening group than in the non-screening group. In addition, interval cancers (IBCs) were more likely than screen-detected cancers (SBCs) to be of HER2-enriched and triple-negative subtype. To our knowledge, this is the first to report detection rate by molecular subtype of breast cancer screening program in a large multi-center population-based dataset in China. The results suggest that we need to further improve the diagnostic yield especially in interval cancers. Our study underscores the urgency to increase breast cancer awareness and early detection in China.

In our study, the overall participation rate among the high-risk women was different (58.19%) from other studies 47.27%-48.2% (Guo et al. 2021; Zhang et al. 2021), but higher than the overall participation in China (40.3%). Publicity and education, mobilization organizations, health awareness of residents, service capabilities of hospitals, and communities all contributed to different participation by regions. Smoking, alcohol consumption, a family history of breast cancer, and benign breast diseases have been confirmed for breast cancer risk factors (Sun et al. 2017). This research discovered that individuals with these characteristics were more likely to engage in breast cancer screening. Women with family history and benign diseases may have more health-oriented consciousness, and more likely to have routine health screening (Li et al. 2020). In addition, we found that participation rates were lower among women aged 70–74 years and with lower education. A lack of awareness and understanding regarding breast cancer screening may be a potential cause. In one meta-analysis from 29 studies, non-participation in screening was associated with low education (Ding et al. 2022). Therefore, targeted education interventions for awareness and cancer prevention are urgently needed in areas with lower screening rates, such as rural communities.

The overall breast cancer detection rates in the screening and non-screening group were at 0.40% and 0.28%, respectively. However, the detection rate was lower than other countries, for example, the United States (0.56%), the Netherlands (0.6%), and Japan (0.5%) (Barlow et al. 2020; Luiten et al. 2020; Ohuchi et al. 2016). It may be due to the low participation of screening and the insufficient follow-up time in our research. In screening group, one-third of breast cancers were IBCs. The incidence and proportion of IBCs may differ based on age and the length of screening interval (Houssami 2017). Our results were similar to the Flemish Breast Cancer Screening Program, with 67% of SBC and 33% of IBC (Timmermans et al. 2017). The results showed that the incidence of IBCs is significantly higher in women aged 50–54 as compared to older women, while the incidence of SBCs is significantly higher in women aged 60–64. A recent study observed that young age of diagnosis was associated with worse survival and more aggressive clinicopathologic features (Timmermans et al. 2017). Therefore, more attention should be paid to and strengthening preventive screening in young women less than 55 years.

The essence of cancer screening is early diagnosis and early treatment of cancer to reduce mortality. We observed a higher detection rate of early stage cases in screening group than in non-screening group, in accordance with the previous reports (Zhang et al. 2021; Huang et al. 2021). A randomized controlled trial in Japan showed that the screened population had 71.3% of cases in stage 0–I, while the non-screened population had 52.0% of cases in stage 0–I (Ohuchi et al. 2016). One population-based breast cancer study in Sweden indicated that screening reduced the risk of advanced breast cancer by 25% in screened population (Duffy et al. 2020). A cohort study for 6396 women aged 50–65 in New South Wales showed that SBCs were more likely to be diagnosed with localized disease (64.1% vs. 48.1%), compared with non-SBCs (Woods et al. 2016). Differences in stage distribution may partly be explained the better survival of SBCs.

Our research supports prior studies which indicate that there was a noticeably higher proportion of luminal A subtype in SBCs (Sihto et al. 2008; Kobayashi et al. 2017), and IBCs were associated with poor tumor characteristics (O'Brien et al. 2018; Defossez et al. 2018). In this study, we found that luminal A subtype was more prevalent in screening women than non-screening ones. A study of 4559 patients in a Chilean cohort reported that the proportion of stage I and "luminal" subtype were significantly higher in SBCs than non-SBCs (Walbaum et al. 2021). One Canadian population-based screening program discovered that IBCs were more probable to present as ER negative compared to SBCs (OR, 2.88; 95% CI 2.01–4.13) (Niraula et al. 2020). The underrepresentation of triple-negative and HER2-enriched subtypes in SBCs is expected as these tumors grow rapidly and thus have shorter preclinical phases. As a result, they are more likely to become symptomatic between scheduled breast cancer screenings (Farshid and Walters 2018). Our study shows that conventional screening is more likely to detect indolent cancer types than fatally aggressive ones. Improvement of diagnostic yield of interval cancers requires personalized screening strategies based on baseline risks in breast cancer screening.

Our study has some limitations. First, the participants were recruited from four urban areas, where healthcare was fairly accessible. Therefore, this study population may not be represent the entire population of Hebei Province. Second, while detailed epidemiological information was collected in a standardized manner by trained study staff, smoking and drinking status were self-reported, which may have led to misclassification. Third, outcome information for breast cancer patients is still being obtained through ongoing follow-up work. Further studies are needed to evaluate the screening effect on breast cancer mortality.

## Conclusions

In summary, in this large-scale screening program, breast cancer screening participation rates were affected by age, education level, postmenopausal status, smoking, drinking, benign breast disease, and family history of breast cancer. We illustrated higher detection rate for both early stage cases and luminal A subtype in screening group than non-screening group. Women who participated in population screening and had interval cancers had a worse subtype and stage distribution. Our results indicate that we need to improve the diagnostic yield, especially in interval cancer, in the future. These findings will provide data support for optimizing population-based breast cancer screening practices in China.

## Data Availability

The datasets generated during and/or analyzed during the current study are available from the corresponding author on reasonable request.
